# Synergistic cytoprotection by co-treatment with dexamethasone and rapamycin against proinflammatory cytokine-induced alveolar epithelial cell injury

**DOI:** 10.1186/s40560-019-0365-5

**Published:** 2019-02-07

**Authors:** Ken Kuwajima, Kyungho Chang, Ai Furuta, Masahiko Bougaki, Kanji Uchida, Shigehito Sawamura, Yoshitsugu Yamada

**Affiliations:** 1 Department of Anesthesiology, Graduate School of Medicine, University of Tokyo, Tokyo, Japan; 2 Anesthesiology and Intensive Care Unit, Teikyo University School of Medicine, Tokyo, Japan

**Keywords:** Acute lung injury, Sepsis, Proinflammatory cytokine, Dexamethasone, Rapamycin, Apoptosis, Cytoprotective, Akt, c-Jun, Synergism

## Abstract

**Background:**

One of the main pathophysiological manifestations during the acute phase of sepsis is massive production of proinflammatory mediators. Clinical trials involving direct suppression of inflammatory mediators to relieve organ dysfunction in sepsis have been extensively performed; however, the clinical outcomes of such trials remain far from satisfactory. Given the need for better sepsis treatments, we have screened various agents with anti-inflammatory properties for cytoprotective effects. In this study, we identified dexamethasone and rapamycin as clinically applicable candidates with favorable synergistic effects against inflammatory cytokine-induced cytotoxicity in vitro and further explored the molecular mechanisms underlying the augmented cytoprotective effects exerted by co-treatment with both drugs.

**Methods:**

Human alveolar epithelial cell-derived A549 cells were stimulated with a mixture of inflammatory cytokines, TNF-alpha, IL-1beta, and IFN-gamma, which induce cellular injury, including apoptosis. This in vitro model was designed to simulate acute lung injury (ALI) associated with sepsis. The cells were co-treated with dexamethasone and rapamycin under cytokine stimulation. Conditioned medium and cell lysates were subjected to further analysis.

**Results:**

Either dexamethasone or rapamycin significantly attenuated cytokine-induced cytotoxicity in A549 cells in a dose-dependent manner. In addition, the simultaneous administration of dexamethasone and rapamycin had a synergistic cytoprotective effect. The applied doses of dexamethasone (10 nM) and rapamycin (1 nM) were considerably below the reported plasma concentrations of each drug in clinical setting. Interestingly, distinct augmentation of both of c-Jun inhibition and Akt activation were observed when the cells were co-treated with both drugs under cytokine stimulation.

**Conclusions:**

A synergistic protective effect of dexamethasone and rapamycin was observed against cytokine-induced cytotoxicity in A549 cells. Augmentation of both of c-Jun inhibition and Akt activation were likely responsible for the cytoprotective effect. The combined administration of anti-inflammatory drugs such as dexamethasone and rapamycin offers a promising treatment option for alveolar epithelial injury associated with sepsis.

**Electronic supplementary material:**

The online version of this article (10.1186/s40560-019-0365-5) contains supplementary material, which is available to authorized users.

## Introduction

To mitigate hyper-inflammatory conditions, such as “cytokine storms” described during sepsis, clinical trials targeting inflammatory mediators have been extensively performed [1, 2]. Although the clinical outcomes of such trials remain far from satisfactory, it should be underlined that these inflammatory mediators can directly activate cellular apoptotic signal pathways [3], corresponding to the assumption that the sepsis-related organ dysfunction is closely associated with cellular apoptosis [4–6]. We believe that because the core pathophysiology of sepsis is defined as an exaggerated host response to microorganisms [7], anti-inflammation strategy, inhibiting the effect of inflammatory mediators, especially proinflammatory cytokines, may still be a promising treatment alternative for sepsis.

We previously reported [3] that a mixture of proinflammatory cytokines (TNF-α/IL-1β/IFN-γ) exerted synergistic cytotoxic effects, including apoptosis and that anti-inflammatory agents or anti-oxidants exerted significant cytoprotective effects against cytokine-induced cytotoxicity in A549 human lung adenocarcinoma cell line [8]. During the course of evaluating the clinical applicability of these agents, we noted that combining the drugs could improve their cytoprotective effects. In the current management for patients with severe sepsis or septic ALI, most of the recommended strategies are just supportive and very few agents possessing direct anti-inflammatory properties have been established as therapeutic drugs [9]. In this study, we evaluated the combination of dexamethasone [10] and rapamycin [11, 12] as clinically applicable candidates with favorable synergistic effects in vitro. Furthermore, we explored the molecular mechanisms underlying these cytoprotective effects against inflammatory cytokine-induced cytotoxicity in A549 cells.

## Methods

### Cell culture and reagents

Human lung carcinoma type 2 epithelium-like A549 cells, purchased from Riken BioResource Center Cell Bank (Tsukuba, Ibaraki, Japan), were grown in Dulbecco’s modified Eagle’s medium (DMEM; Sigma-Aldrich Life Sciences, MO, USA) with 10% heat-inactivated fetal bovine serum (FBS; GE Healthcare Japanhealth, Tokyo, Japan), 100 U/ml penicillin, 100 mg/ml streptomycin in 10 cm dishes at 37 °C in a humidified atmosphere of 5% CO2. A549 cell line has been characterized as an in vitro model of alveolar type 2 pneumocytes of the human lung because of capacity of secreting lung surfactant-associated glycoproteins [8].

Reagents were purchased commercially as follows: human IL-1β (Humanzyme, Chicago, IL, USA); human TNF-α (Prospec, Rehovot, Israel), human IFN-γ (Peprotech, NJ, USA); dexamethasone, Fas ligand (super Fas Ligand) (Enzo, PA, USA); RIPA buffer (Thermo Fischer Scientific, Tokyo, Japan); lipopolysaccharide (LPS: *E. coli* O111:B4, Sigma-Aldrich Life Sciences); rapamycin, parthenolide, SP600125, SB203580, U0123, LY294002, and QVD-OPh (Cayman Chemical, MI, USA).

Antibodies were as follows: anti-iNOS (R&D systems, MN, USA); anti-HO-1 (Enzo); anti-beta actin (MBL, Nagoya, Japan); anti-JNK (Abcam Japan, Tokyo, Japan); anti-cox-2 (BD Japan, Tokyo, Japan); anti-ICAM-1 (Santa Cruz Biotechnology, TX, USA). All of the other antibodies were purchased from CST Japan, Tokyo, Japan.

### Cell treatment

A549 cells were seeded in culture plates at 10^5^ cells/cm^2^ and cultured overnight. After cell attachment, the concentration of FBS in medium was decreased from 10 to 2% by medium change. In pretreatment experiments, cells were incubated with intervening agents (dexamethasone, rapamycin, parthenolide, SB600125, and QVD-OPh) for 1 h and then stimulated with proinflammatory cytokines (TNF-α/IL-1β/IFN-γ; the Cytokine Mixture, designated as CM in the figures). Intervening agents were dissolved in ethanol or dimethyl sulfoxide (DMSO), and cytokines were dissolved in 0.1% bovine serum albumin (BSA) solution. Detailed ways of administration such as time points were also mentioned in each figure legend.

To simulate the complex inflammatory environment in the alveolar space in sepsis-associated acute lung injury (ALI), these representative proinflammatory cytokines were selected. The combination of TNF-α/IL-1β/IFN-γ (found as “cytomix” in the key word search list in PubMed [13]) is widely accepted as a way of simulating a hyperinflammatory status in vitro. The cytokines were mainly used at a concentration of 10 ng/mL in the present study, as in our previous study [3]. Control cells were treated with the corresponding vehicle (ethanol or DMSO) alone. The concentration of vehicle in the medium was maintained at < 0.2% to minimize the effect of solvents. In some experiments, to check the efficacy of post-treatment of cytoprotective agents (dexamethasone or rapamycin), they were added to culture cells after cytokine stimulation at the indicated times.

### Western blotting

A549 cells were harvested 24–60 h after cytokine stimulation depending on the parameters of the experiment. After washing with phosphate-buffered saline (PBS), cells were directly lysed in 2 × sodium dodecyl sulfate(SDS) sample buffer without bromophenol blue dye (100 mM Tris-HCl, pH 6.8, 4% SDS, 20% glycerol, 2% β-mercaptoethanol, 25 mM ethylenediaminetetraacetic acid (EDTA)). Protein concentration was determined using a protein quantification assay kit (Macherey-Nagel, Duren, Germany). Equal amounts of protein were separated by SDS-polyacrylamide gel electrophoresis (SDS-PAGE) and then transferred onto nitrocellulose membranes. After blocking with 2% ECL advance blocking reagent™ (GE Healthcare, NJ, USA) in PBS containing 0.1% Tween 20 (polyoxyethelenesorbitan monolaurate), the membranes were incubated overnight with primary antibodies at 4 °C. After washing and incubation with horseradish peroxidase (HRP)-conjugated anti-rabbit or anti-mouse IgG, the antigen-antibody complexes were detected using chemiluminescence (ECL select detection system™, GE Healthcare) and then visualized using an Imagequant Las4000 system™ (Bio-Rad, CA, USA).

### Evaluation of overall cytotoxicity of cytokines in A549 cells

A549 cells were seeded in 24-well culture plates at 5 × 10^4^ cells/well. The cytotoxic effects of cytokines in A549 cells were evaluated after 48–60 h of cytokine stimulation depending on the parameters of the experiment. Cytotoxicity was evaluated quantitatively by monitoring lactate dehydrogenase (LDH) concentration in culture medium.

LDH concentration in culture medium was determined using Cytotoxicity Detection Kit Plus™ (Roche Applied Science, Mannheim, Germany) according to the manufacturer’s instructions with some modifications. Briefly, first medium was collected as “released sample” from each well. Then, residual cells in the well were completely lysed with lysis buffer. After removing cell debris by centrifugation, second medium was collected as “total sample.” LDH activity in each sample was determined by measuring absorbance with a spectrophotometer at a wavelength of 490 nm (with a reference wavelength of 620 nm). After subtracting the background control value (2% FBS medium only), cytotoxicity was expressed as a ratio (“released value” divided by “total value”).

### Quantitative evaluation of caspase activity

To explore the contribution of apoptosis to the cytokine-induced cytotoxicity in A549 cells, caspase activity was quantitatively measured using culture medium as with LDH measurements. The levels of soluble caspase-cleaved cytokeratin 18 fragments were measured by M30 Cytodeath™ Enzyme-Linked Immunosorbent Assay (ELISA) (Peviva, Bromma, Sweden) according to the manufacturer’s instructions [3]. The M30 antibody recognizes a neo-epitope (Asp396) that is exposed after cleavage of cytokeratin 18 by effector caspases (caspase 3, 6, and 7) in human, monkey, and bovine epithelial cells. Each sample value was presented relative to the untreated control value, which was tentatively named as “apoptosis index” in the figures. Apoptosis was also evaluated by western blotting using anti-cleaved caspase 7 or anti-cleaved poly ADP-ribose polymerase (PARP) antibodies.

### In vivo endotoxemic mouse model

Animal studies were conducted using procedures approved by the animal experimentation committee of the Graduate School of Medicine, the University of Tokyo, Tokyo, Japan (Med-P13-108). Male C57BL/6 mice (10 weeks; Nippon Bio-Supp. Center, Tokyo, Japan) received intraperitoneal injections with saline (control) or LPS (10 mg/kg), generating an in vivo endotoxemic model, which simulated temporal manifestation of hypercytokinemia [14]. Twenty-four hours after drug injection, vital organs (heart, kidney, liver, and lung) were collected under sevoflurane-anesthesia and flash-frozen with liquid nitrogen. Organ tissues were pulverized while frozen and lysed in RIPA buffer. Total cell lysates were then subjected to western blotting. (The result was presented in the Additional file 1: Figure S1).

### Statistical analysis

Experiments that required statistical analysis were carried out in triplicate at least and repeated to confirm representative results. Data are expressed as mean ± standard error of the mean (SEM). Statistical significance of differences between means was determined by analysis of variance (ANOVA) followed by post hoc Tukey’s test for multiple comparisons using Statmate software (ATMS, Tokyo, Japan). In all analyses, *P* < 0.05 was considered to indicate statistical significance.

## Results

### Combination of proinflammatory cytokines markedly affected inflammatory protein expression in A549 cells

In our previous study [3], we confirmed that apoptotic pathways were activated when human alveolar epithelial cell-derived A549 cells were stimulated with proinflammatory cytokines. To further elucidate the origin of cytotoxicity induced by proinflammatory cytokine, we evaluated the detailed profile of inflammatory protein expressions stimulated with these cytokines in this study.

**Fig. 1:**
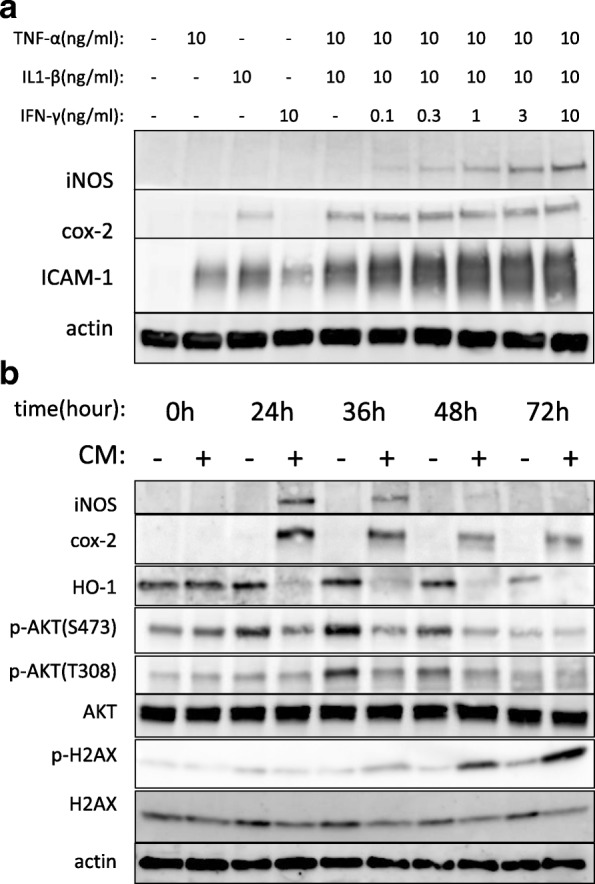
The effects of proinflammatory cytokines on protein expression in A549 cells. **a** Combinations of proinflammatory cytokines significantly augmented inflammation-associated protein expression in A549. A549 cells were stimulated with the indicated cytokines. Twenty-four hours after cytokine stimulation, total cell lysate was collected and then subjected to western blotting. iNOS and ICAM-1 expression were dose-dependently augmented by IFN-γ added with TNF-α/IL-1β. Cox-2 expression was augmented when TNF-α was added with IL-1β. **b** The temporal changes in inflammation-associated protein expression induced by cytokines (cytokine mixture, CM) in A549 cells. Total cell lysate was collected at the indicated time points after CM stimulation and then subjected to western blotting using the indicated antibodies. iNOS and cox-2 induction peaked at 24 h after CM stimulation and gradually decreased thereafter. HO-1 disappeared 24 h after CM stimulation. Phosphorylation of Akt (p-Akt) decreased gradually as time went by both in T308 and S473, while phosphorylation of H2AX (p-H2AX) became prominent 48 h after CM stimulation. Cytokine mixture (CM): TNF-α(10 ng/ml), IL-1β(10 ng/ml), and IFN-γ(10 ng/ml)

As shown in Fig. 1a, inducible nitric oxide synthase (iNOS) and intracellular adhesion molecule-1 (ICAM-1) expression were augmented by IFN-γ added to TNF-α/IL-1β in a dose-dependent manner. Cyclooxygenase-2 (cox2) expression was augmented when TNF-α was added with IL-1β. This implies that inflammatory cytokines worked synergistically and that IFN-γ might also play an indispensable role in clinical inflammation with TNF-α and IL-1β.

Next, we observed the temporal changes in inflammation-associated protein expression induced by the cytokine mixture in A549 (Fig. 1b). iNOS and cox-2 induction peaked at 24 h after cytokine stimulation and gradually decreased thereafter. Heme oxygenase-1 (HO-1), a transcription factor that activates a broad range of anti-oxidant enzymes [15], disappeared 24 h after cytokine stimulation. Basal activity of Akt, a representative member of survival signal pathways [16], decreased gradually during the time course, measured by phosphorylation of both T308 and S473, while phosphorylation of histone H2AX, which is an indicator of DNA instability [17] became prominent 48 h after cytokine stimulation. Because HO-1 and Akt are beneficial for cell survival, decreased levels or inactivation of these molecules might contribute to cytokine-induced cytotoxicity.

### Caspase inhibition only partially attenuated cytokine-induced cytotoxicity, while it almost completely blocked Fas ligand-induced cytotoxicity

**Fig. 2:**
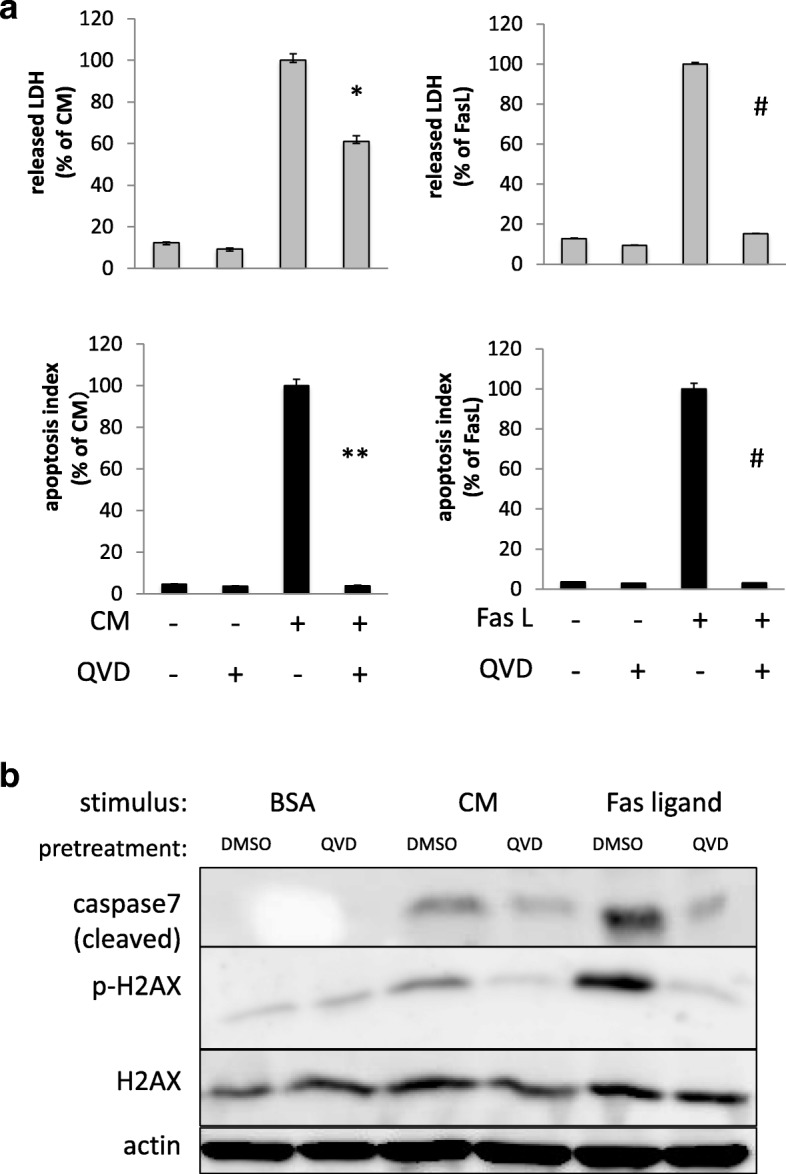
Caspase inhibition only partially attenuated cytokine-induced cytotoxicity, while it almost completely blocked Fas ligand-induced cytotoxicity. A549 cells were pretreated with DMSO (vehicle) or QVD-OPh (pan-caspase inhibitor) 1 h before CM or Fas ligand (Fas L; specific apoptosis inducer) stimulation. Forty-eight hours after each stimulation, cytotoxicity and apoptosis were evaluated as described in the “Methods” section. **a** Grey bar graph shows cytotoxicity evaluated by monitoring the concentration of released LDH in culture medium as described in the “Methods” section. Black bar graph shows caspase activation estimated by determining the concentration of cleaved cytokeratin 18 in culture medium using an M30 cytodeath ELISA kit as described in the “Methods” section. QVD-OPh only partially blocked CM-induced cytotoxicity (determined by LDH), although it almost completely blocked the apoptotic component (termed as “apoptotic index”) estimated by cleaved cytokeratin18. On the other hand, LDH increase by specific apoptotic pathway stimulation (using Fas ligand; Fas L) was almost completely inhibited by QVD-OPh as well as cleavage of cytokeratin18. Bar graph shows means ± SEM. *P < 0.05 vs. CM alone, **P < 0.01 vs. CM alone, ^#^*P* < 0.01 vs. Fas L alone. **b** Total cell lysates were collected and then subjected to western blotting. Caspase 7 cleavage and phosphorylation of histone H2AX were significantly blocked by QVD-OPh pretreatment in CM stimulation as well as Fas ligand stimulation

Because cytotoxicity is closely linked to cellular apoptosis, next we investigated whether direct inhibition of apoptosis through a caspase inhibitor could suppress cytokine-induced cytotoxicity. A549 cells were pretreated with DMSO (vehicle) or a pan-caspase inhibitor (QVD-OPh) 1 h before stimulation with cytokines or apoptosis inducing Fas ligand. Forty-eight hours after stimulation, cytotoxicity was evaluated as described in the “Methods” section. Unexpectedly, as shown in Fig. 2a, caspase inhibition by QVD-OPh only partially blocked cytokine-induced cytotoxicity, as determined by LDH levels. However, it almost completely blocked apoptosis, as determined by measuring cleaved CK18 by M30 ELISA (apoptosis index) (Fig. 2a) and cleaved caspase 7 levels by western blotting (Fig. 2b). On the other hand, both of LDH release and apoptosis triggered by selective apoptotic pathway stimulation, using Fas ligand, were almost completely inhibited by QVD-OPh (Fig. 2a, b). These results suggested that signal transduction activated by inflammatory cytokines was not straightforward and encouraged us to further explore the details of cytotoxic signal pathways evoked by inflammatory cytokines from the early phase in the response.

### Cytotoxicity induced by proinflammatory cytokines was partially attributable to JNK/c-Jun signal pathway

**Fig. 3:**
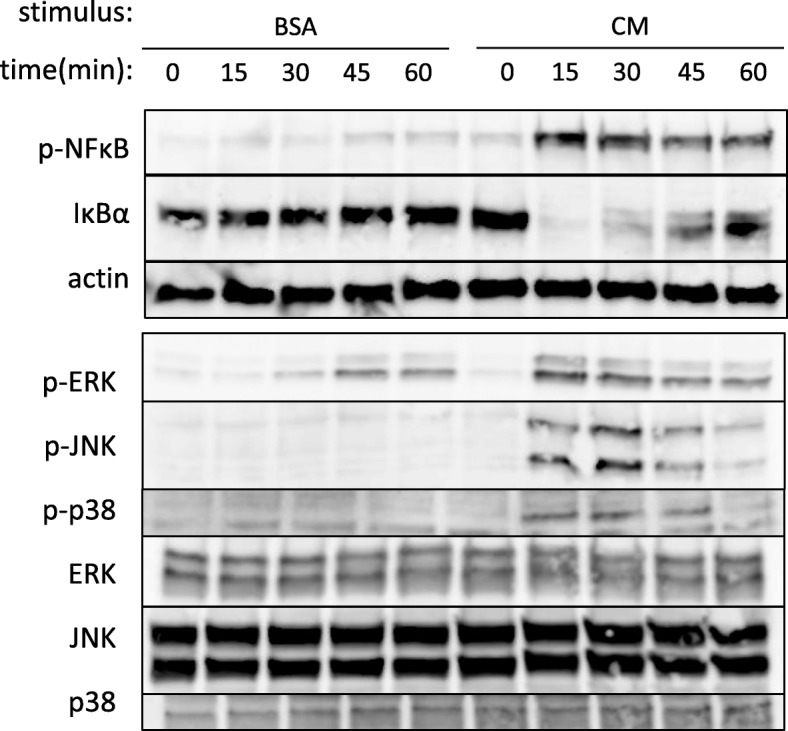
Cytokines activated the NF-κB and MAP kinase pathways in A549 cells. A549 cells were stimulated with BSA (vehicle control) or CM at time 0. Total cell lysates were collected at the indicated time points and then subjected to western blotting using the indicated antibodies. Upper panel shows the phosphorylation of NF-κB (p-NFκB) peaked at 15 min after CM stimulation and sustained at least for 1 h. IκBα rapidly degraded within 15 min after CM stimulation and recovered gradually. Lower panel shows the maximum phosphorylation was observed between 15 min and 30 min for ERK, JNK, and p38 after CM stimulation. Weak activation of ERK was also observed after vehicle (BSA) treatment alone. The total protein amounts of each MAP kinase were unchanged during the observed period

The transcription factor NF-κB is a central mediator of the inflammatory response [18]. Firstly, we checked for activation of the NF-κB pathway after cytokine stimulation in A549 cells. As shown in Fig. 3 (upper panel), IκBα, which sequesters NF-κB in the cytosolic compartment in the non-stimulated state, was rapidly degraded within 15 min after cytokine stimulation and reappeared after 45 min. Activation of NF-κB, confirmed by phosphorylation of NF-κB, also occurred within 15 min and lasted for at least 1 h. Similar to NF-κB, MAP kinases are major stress-activated signal molecules and are activated during inflammation [18]. As shown in Fig. 3 (lower panel), the three major MAP kinases (ERK, JNK, and p38) were promptly activated after cytokine stimulation. Then, to reveal a contributing role of each signal molecule for cytotoxicity, selective inhibitors were pretreated before cytokine stimulation.

**Fig. 4:**
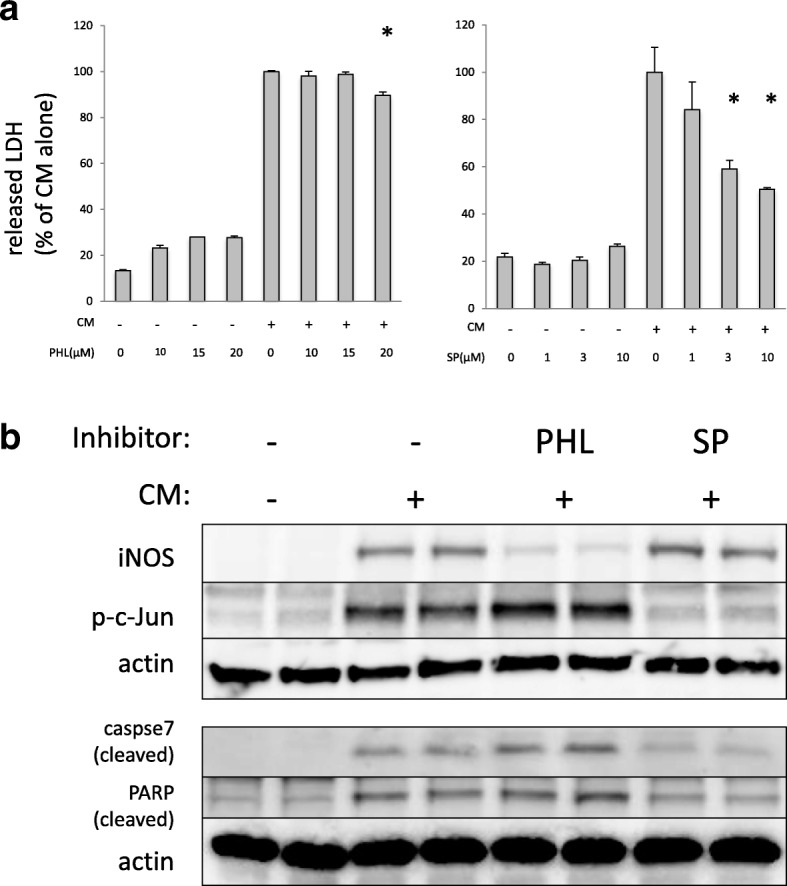
The effect of an NF-κB inhibitor (parthenolide) or JNK inhibitor (SP600125) on cytokine-induced cytotoxicity in A549. **a** Dose-response effect of parthenolide (PHL) or SP600125 (SP) on cytokine-induced cytotoxicity in A549. A549 cells were pretreated with parthenolide or SP600125 (SP) at the indicated concentration for 1 h and then stimulated with CM. Forty-eight hours after CM stimulation, cytotoxicity was evaluated by monitoring the concentration of LDH in culture medium as described in the “Methods” section. Parthenolide only slightly attenuated cytotoxicity at the maximum, nearly toxic, dose. While SP600125 suppressed cytokine-induced cytotoxicity in A549 cells in a dose-dependent manner. Bar graph shows means ± SEM. * *P* < 0.01 vs. CM alone. **b** The effects of parthenolide (PHL) and SP600125 (SP) on inflammation-associated protein expression induced by cytokines in A549 cells. Total cell lysate was collected 24 h (upper panel) or 48 h (lower panel) after CM stimulation and then subjected to western blotting using the indicated antibody. Upper panel shows selective inhibition of the NF-κB or JNK pathway was confirmed by iNOS expression or phosphorylated c-Jun (p-c-Jun), respectively. Lower panel shows SP600125(3 μM) markedly inhibited cleavage of caspase 7 and PARP, while parthenolide(15 μM) did not, although it blocked iNOS expression with this concentration

As shown in Fig. 4a, parthenolide, an inhibitor of the NF-κB, slightly attenuated cytotoxicity at the maximum, nearly toxic, dose. While JNK inhibitor, SP600125 suppressed cytokine-induced cytotoxicity in A549 cells in a dose-dependent manner. ERK inhibitor (U0123) and p38 inhibitor (SB203580) did not rescue cytotoxicity (data not shown). As shown in Fig. 4b, parthenolide failed to inhibit caspase activation while inhibiting iNOS induction. SP600125 effectively inhibited caspase/PARP activation and c-Jun phosphorylation. Because NF-κB activates both of inflammation-associated apoptotic and survival pathways, the effect of direct inhibition of NF-κB pathway might be unpredictable. On the other hand, JNK pathway inhibition may be much more important for attenuating cytokine-induced cytotoxicity in this context.

### Simultaneous administration of dexamethasone and rapamycin had a synergistic protective effect against cytokine-induced cytotoxicity

**Fig. 5:**
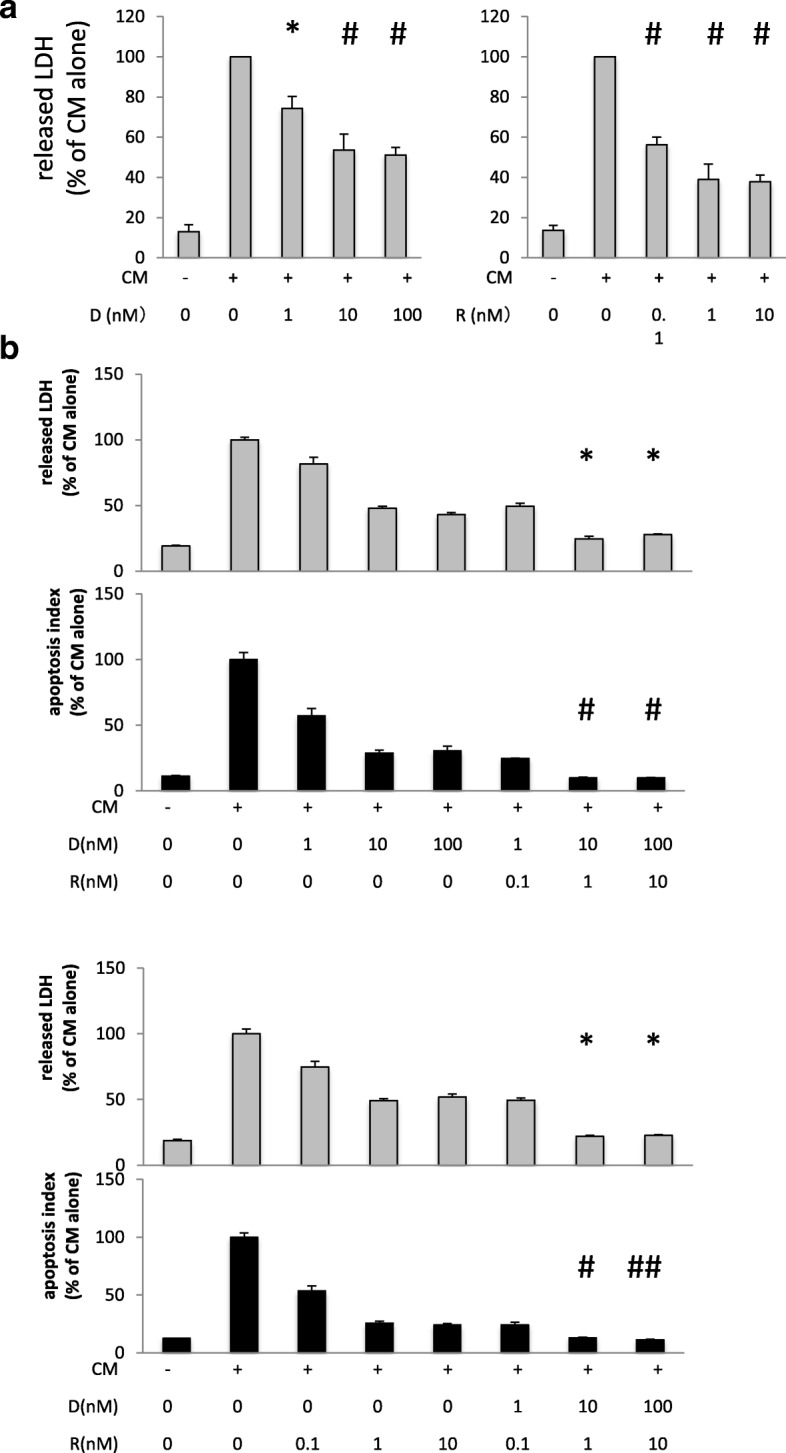
Synergistic effect of dexamethasone and rapamycin against cytokine-induced cytotoxicity in A549. **a** Dose-response effect of dexamethasone (D) or rapamycin (R) against cytokine-induced cytotoxicity in A549. A549 cells were pretreated with dexamethasone or rapamycin at the indicated concentration for 1 h and then stimulated with CM. Forty-eight hours after CM stimulation, cytotoxicity was evaluated by monitoring the concentration of LDH in culture medium as described in the “Methods” section. Both dexamethasone (D) and rapamycin (R) significantly attenuated CM-induced cytotoxicity in A549 cells in a dose-dependent manner. Bar graph shows means ± SEM. *P < 0.05 vs. CM alone, ^#^ *P* < 0.01 vs. CM alone. **b** Synergistic effect of dexamethasone (D) and rapamycin (R) against cytokine-induced cytotoxicity and apoptosis in A549. Grey bar graph shows cytotoxicity evaluated by monitoring the concentration of released LDH in culture medium as described in the “Methods” section. Bar graph shows means ± SEM. Black bar graph shows caspase activation estimated by determining the concentration of cleaved cytokeratin 18 in culture medium using an M30 cytodeath ELISA kit as described in “Methods”. Bar graph shows means ± SEM. Upper pair of the graphs shows the effect of combined treatment compared with that of dexamethasone alone. *P<0.01 vs. D10/R0, D100/R0, ^#^*P* < 0.05 vs. D10/R0, D100/R0. Lower pair of the graphs shows the effect of combined treatment compared with that of rapamycin alone. *P < 0.01 vs. R1/D0, R10/D0 #; *P* < 0.05 vs. R1/D0 ^##^*P* <0.05 vs. R1/D0, R10/D0

**Fig. 6:**
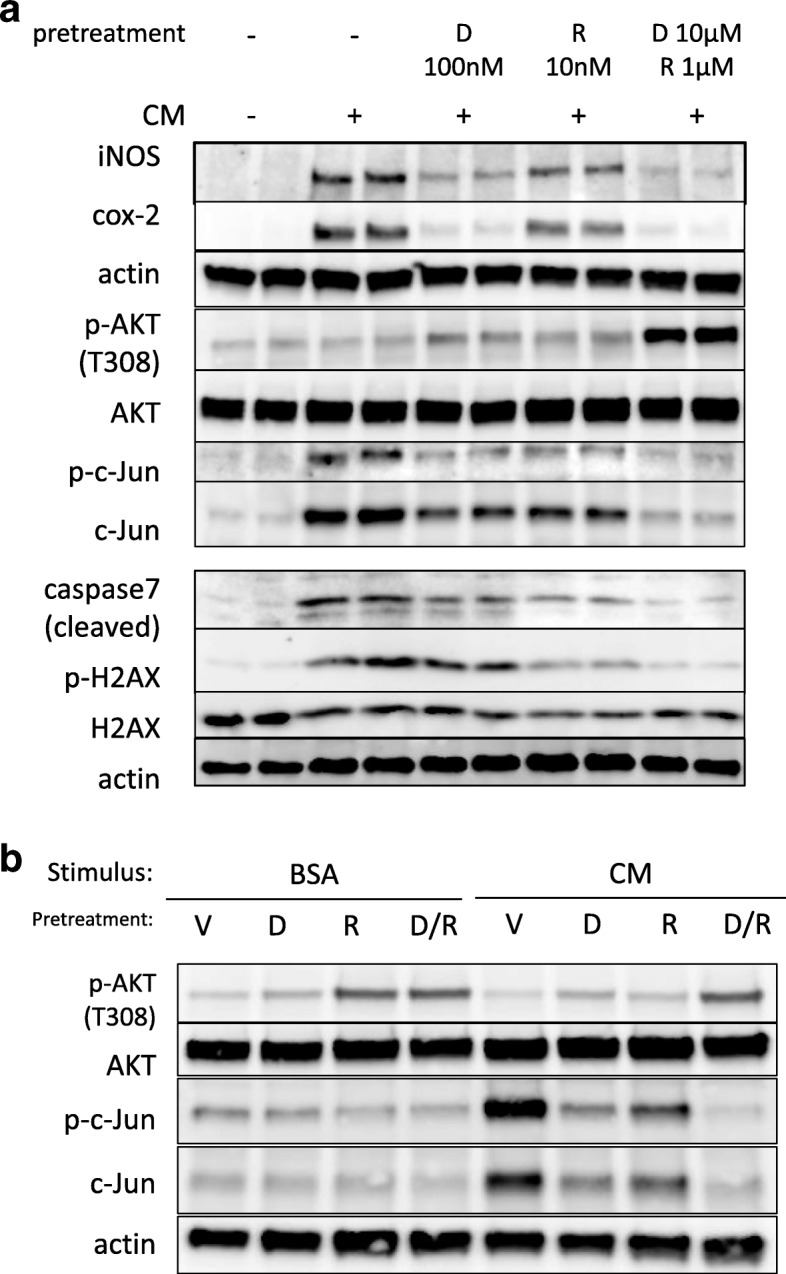
The effect of dexamethasone or rapamycin on phosphorylation of Akt or c-Jun. **a** A549 cells were pretreated with dexamethasone (D) or rapamycin (R) at the indicated concentration for 1 h and then stimulated with CM. Total cell lysate was collected 24 h (upper panel) or 48 h (lower panel) after CM stimulation and then subjected to western blotting using the indicated antibody. The effect of combination of dexamethasone (10 nM) and rapamycin (1 nM) surpassed that of 10 times concentration of each drug in terms of the inhibition of iNOS, cox2, cleaved caspase 7, and phosphorylated H2AX. In addition, both of augmentation of phosphorylated Akt (p-Akt) and inhibition of phosphorylated c-Jun (p-c-Jun) were remarkable in combined treatment of dexamethasone and rapamycin. **b** A549 cells were pretreated with dexamethasone (10 nM, D), rapamycin (1 nM, R) or both (10 nM D/1 nM R) for 1 h and then stimulated with BSA (vehicle control) or CM. Total cell lysate was collected 24 h after stimulation and then subjected to western blotting using the indicated antibody

Although JNK inhibitor SP600125 had potential to rescue cytokine-induced cytotoxicity, no selective JNK inhibitors are clinically applicable at the present. Then, we turned to focus on two anti-inflammatory agents, dexamethasone, and rapamycin. They are clinically applicable agents presently and found to have cytoprotective effects against cytokine-induced cytotoxicity in our preliminary experiments and reports by other investigators [19, 20]. We investigated their appropriate doses, mechanism of cytoprotection including JNK pathway inhibition, and synergism by co-treatment. As shown in Fig. 5a, either dexamethasone or rapamycin significantly attenuated the overall cytokine-induced cytotoxicity in A549 cells in a dose-dependent manner. According to Fig. 5a, doses exerting sub-maximal effects were 10 nM in dexamethasone and 1 nM in rapamycin. Previous studies suggest that the presumed clinical serum concentration of dexamethasone [21] and rapamycin [22] is around 100 nM and 10 nM, respectively. We chose our doses by using one-tenth of these reported concentrations, 10 nM dexamethasone, and 1 nM rapamycin, expecting fewer adverse effects when clinically applied. As shown in Fig. 5b, combined administration of dexamethasone and rapamycin showed a profound protective effect by measuring LDH levels and apoptosis (apoptosis index). In addition, the synergistic effects by co-treatment of dexamethasone and rapamycin were also observed in the inhibition of caspase 7 and phosphorylation of histone H2AX as well as the inhibition of iNOS and cox-2 induction (Fig. 6a). Therefore, co-treatment with 10 nM dexamethasone and 1 nM rapamycin improved a broad range of biological effects compared with that of single treatment of 100 nM dexamethasone or 10 nM rapamycin alone.

### Distinct augmentation of c-Jun inhibition and Akt activation was observed when dexamethasone and rapamycin were co-treated under cytokine stimulation

Next, we explored the underlying molecular mechanism for the cytoprotective effects observed from the combined treatment with dexamethasone and rapamycin. Since cytokine stimulation gradually inhibited Akt activation (Fig. 1b) and JNK inhibition suppressed cytotoxicity in a dose-dependent manner (Fig. 4a), we expected that these two pathways were relevant. As shown in Fig. 6a, distinct augmentation of c-Jun inhibition and Akt activation was observed when dexamethasone and rapamycin were used together before cytokine stimulation. In addition, we measured the effect of dexamethasone or rapamycin on Akt and c-Jun phosphorylation with or without cytokine stimulation. As shown in Fig. 6b, under BSA stimulation (“without cytokine stimulation”), rapamycin treatment alone increased Akt phosphorylation and decreased c-Jun phosphorylation, but these effects were not prominent under cytokine stimulation. Instead, adding dexamethasone on rapamycin treatment maintained the effects that rapamycin triggered under cytokine stimulation.

### Dexamethasone and rapamycin still exerted synergistic cytoprotection with post-treatment after challenge of cytokines in A549 cells

Lastly, cytoprotective effects of dexamethasone and rapamycin were tested as clinical therapeutic agents, meaning that drug administration began after cytokine challenge in A549 cells.

**Fig. 7:**
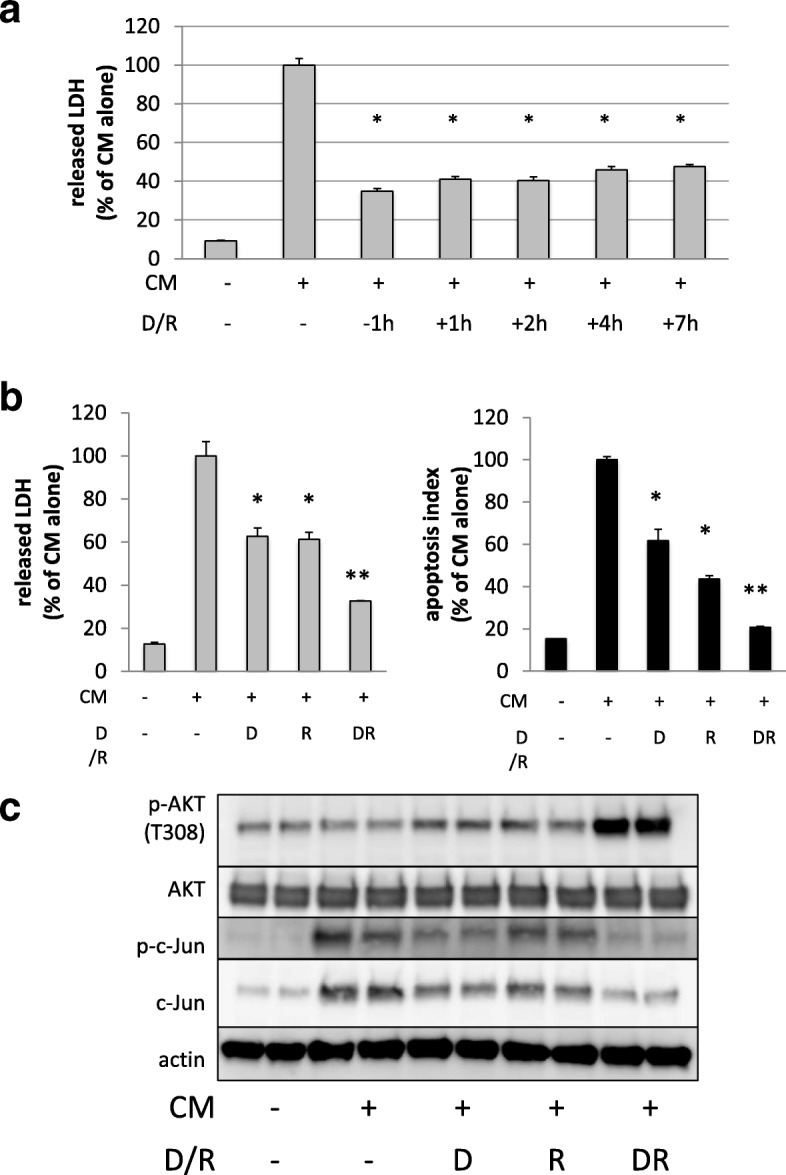
Dexamethasone and rapamycin still exerted synergistic cytoprotection with post-treatment after challenge of cytokines in A549 cells. **a** A549 was stimulated with the cytokine mixture (CM). Co-treatment of dexamethasone (D, 10 nM) and rapamycin(R, 1 nM) was initiated 1h before (− 1 h), 1 h after (+ 1 h), 2 h after (+ 2 h), 4 h after (+ 4 h), 7 h after (+ 7 h) cytokine stimulation, and released LDH was evaluated 48 h after cytokine stimulation as described in the “Methods” section. Post-treatment of dexamethasone and rapamycin still inhibited LDH release significantly at least up to 7 h after cytokine challenge, although the degree of cytoprotection gradually decreased compared to pre-treatment (− 1 h). Bar graph shows means ± SEM. *P < 0.01 vs. CM alone. **b** A549 cells were treated with ethanol (vehicle) or dexamethasone (D, 10 nM), rapamycin (R, 1 nM) or both (DR) 2 h after cytokine stimulation. Forty-eight hours after cytokine stimulation, cytotoxicity (grey bar graph) and apoptosis (black bar graph) were evaluated as described in the “Methods” section. Bar graph shows means ± SEM. *P < 0.01 vs. CM alone, **P < 0.01 vs. CM/D, CM/R. **c** A549 cells were treated with ethanol (vehicle) or dexamethasone (D, 10 nM), rapamycin (R, 1 nM) or both (DR) 2 h after CM stimulation. Total cell lysates were collected 48 h after CM stimulation and then subjected to western blotting using the indicated antibody. Both of augmentation of phosphorylated Akt(p-Akt) and inhibition of phosphorylated c-Jun(p-c-Jun) were still remarkable in post-treatment of both of dexamethasone and rapamycin compared with either dexamethasone or rapamycin treatment

As shown in Fig. 7a, post-treatment of dexamethasone and rapamycin still inhibited LDH release significantly at least up to 7 h after cytokine challenge, although the degree of cytoprotection gradually decreased compared to pre-treatment (− 1 h). Besides, in samples in which dexamethasone and rapamycin were added 2 h after cytokine stimulation, synergistic effects of both drugs were still observed in terms of inhibition of overall cytotoxicity (released LDH) and apoptosis (apoptosis index) (Fig. 7b), and phosphorylation of AKT and c-Jun (Fig. 7c), as in pre-treatment samples. These suggest that there may be therapeutic time window during which pharmacological interventions are possible after massive production of proinflammatory cytokines occur in sepsis-like conditions.

## Discussion

The main findings of the current study can be summarized as follows:

1. A mixture of representative proinflammatory cytokines (TNF-α/IL-1β/IFN-γ) activated NF-κB and MAP kinase pathways, eventually exerting cytotoxicity, including apoptosis, in A549 cells.
2. JNK/c-Jun pathway is, at least partially, responsible for cytokine-induced cytotoxicity.
3. Either dexamethasone or rapamycin significantly attenuated the cytokine-induced cytotoxicity in A549 cells in a dose-dependent manner.
4. Simultaneous administration of dexamethasone and rapamycin had a synergistic cytoprotective effect at considerably lower doses than the presumed clinical serum concentrations of each drug.
5. Distinct augmentation of both of c-Jun inhibition and Akt activation was noted when the cells were co-treated with both drugs under cytokine stimulation.
6. Synergism of dexamethasone and rapamycin was still valid when the two drugs were administered at several hours after cytokine challenge.

### Potential of combined use of anti-inflammatory drugs for ALI-associated sepsis

Because most of anti-inflammatory therapies that target only a single molecule in the inflammatory cascade failed to produce positive results in clinical studies, studying the immunosuppressive status rather than hyper-inflammatory status has become a more promising avenue for developing new treatment strategies for sepsis [23–25]. However, it would be an oversimplification to postulate that anti-inflammation therapies themselves are of no clinical value. In fact, some trial results showed clinical efficacy of anti-inflammation strategies among selected patient populations [26]. Similarly, steroids, the most clinically successful anti-inflammatory agents, have survived as a therapeutic option to treat septic shock according to recent guidelines [9, 19], though they are not solely intended as immunosuppressors. Steroid is not a magic bullet, however, and the efficacy of steroid alone is limited. Sepsis is a highly complex disease state, and it would be difficult to improve clinical outcomes by a single intervention. Thus, our standpoint is that an anti-inflammation strategy should involve combined therapy [1], which is a common clinical practice for treating HIV infection, bacterial infections, and neoplasm. The validity of adding another drug on corticosteroid has been mentioned in recent reports investigating a new strategy for sepsis or acute lung injury [27, 28].

Rapamycin, first discovered as a selective mTOR inhibitor, is another potent, clinically available anti-inflammatory drug and several reports have already demonstrated its efficacy in various proinflammatory diseases [20]. Our data here indicated that combined administration of dexamethasone and rapamycin showed potentiation not only in anti-inflammatory effects, but also in cytoprotective effects. The therapeutic serum concentrations are reported to be approximately 50–100 nM for dexamethasone [21] and 10–15 nM for rapamycin [22]; the drug doses adopted for combination therapy used here were much lower than these concentrations, reducing the adverse effects expected when both drugs are used together.

### Putative molecular mechanism of synergistic cytoprotection induced by co-treatment with dexamethasone and rapamycin under cytokine stimulation

Because a selective JNK inhibitor, SP600125, inhibited cytokine-induced cytotoxicity in a dose-dependent manner, JNK/c-Jun pathway can be a pharmacological target in alleviating inflammation-induced alveolar epithelial injury. It is well established that JNK/c-Jun pathway is strongly associated with apoptosis [29, 30]. In fact, because co-treatment with dexamethasone and rapamycin augmented the inhibition of c-Jun phosphorylation, the synergistic cytoprotective effect would be, at least partially, derived from the inhibition of JNK/c-Jun pathway. Although we could not show here that peak activation of JNK was inhibited by dexamethasone and rapamycin, phosphorylation of c-Jun can be regarded as the result of the sum of upstream JNK activation.

Augmentation of Akt activity by co-treatment with dexamethasone and rapamycin is also a very promising observation. Akt is well known as a primary molecule in survival signal pathways [29, 31], so it is probable that Akt also contributes to cell survival under cytokine stimulation. To clarify this point, we performed an additional experiment, wherein we checked whether the inhibition of Akt using a PI-3K inhibitor, LY294002, could cancel cytoprotection induced by dexamethasone and rapamycin. Unexpectedly, PI3K inhibitor itself exerted cytoprotective effects against inflammatory cytotoxicity, independent of Akt activity (data not shown). This implies that augmented activation of Akt in this context might be triggered by a different kinase other than PI-3K. To clarify the direct protective contribution of Akt, direct inhibition of Akt, such by siRNA knockdown, would be necessary in the further analysis.

### Cell death mechanisms other than apoptosis are involved in cellular dysfunction under hyper-inflammatory conditions

A number of studies argued that apoptosis is closely linked to cytotoxicity evoked by severe inflammation in vivo [5, 6, 32, 33]. Corresponding to these observations, caspase activation and DNA instability were confirmed in vital organs such as the kidney and liver in an endotoxemic mouse which was a commonly used model of hypercytokinemia in vivo (Additional file 1: Figure S1) . Our previous [3] and present data supported that a mixture of just three proinflammatory cytokines directly induced cellular apoptosis without the aid of infiltrating cells such as neutrophils. While, as shown in Fig. 2, direct inhibition of caspases by QVD-OPh (pan-caspase inhibitor), which are downstream of apoptotic signal pathways, did not rescue cytokine-induced cytotoxicity although apoptosis and DNA instability were largely suppressed. These results suggested that cytokine-triggered cytotoxicity is complex and that cell death pathways other than apoptosis become prominent when apoptosis is specifically blocked [32]. In other words, caspase inhibitors can only act downstream of apoptotic signal pathways and cannot inhibit other cytotoxic signals that are already activated upstream in response to proinflammatory cytokines. According to these findings, any effort to pharmacologically counter the cytotoxic effects exerted by proinflammatory cytokines on upstream of the cytotoxic signal pathways would be of clinical importance.

### Limitations

This study has some limitations. First, augmented cytoprotection by dexamethasone and rapamycin was only proved in cultured human cell lines and results of the animal study have not yet been presented. We are now investigating whether synergistic protective effects are observed using the endotoxemic mouse model. Further consideration would be necessary to decide appropriate drug doses and timing of administration. However, publishing current results from in vitro studies would be meaningful to show putative underlying molecular mechanisms responsible for biological effects.

Second, in the present data, we have not demonstrated a direct relationship between augmented Akt activity and cytoprotection in this context. Considering an extensive role of Akt in stress-activated intracellular signal pathways, however, it is unlikely that such an alteration in Akt activity will not cause any other biological effects. Even if direct inhibition of Akt would not prevent synergistic cytoprotection, it would be possible that any kinase situated upstream above Akt might be responsible for cytoprotection. In addition, this is the first study, to our knowledge, that shows that co-administration of dexamethasone and rapamycin drastically augmented Akt activity and it is important to report this drug interaction.

Finally, it might be controversial to determine whether the effect induced by co-treatment with dexamethasone and rapamycin should be expressed as “synergistic” or “additive.” Generally speaking, when the working mechanisms of the two agents are different, the biological effects are expected to be synergistic rather than additive. In this study, because the effects of combined administration of both drugs surpassed the maximum effects of each drug on being used alone and because the signal pathways triggered by each drug seemed different (as shown in Fig. 6b), the word “synergism” was adopted. Further elucidation of activated signal pathways by each drug will confirm this point.

## Conclusions

A synergistic cytoprotective effect of dexamethasone and rapamycin was observed against cytokine-induced cytotoxicity in A549 cells. Augmentation of c-Jun inhibition and Akt activation were inferred to be responsible for the synergistic effects. The combined administration of distinct anti-inflammatory drugs such as dexamethasone and rapamycin offers a promising treatment option for alveolar epithelial injury associated with sepsis.

## Additional file

Additional file 1: **Figure S1.** Changes of inflammation-associated protein expression in an endotoxemic mouse. Male C57BL/6 mice (10 weeks) were challenged intraperitoneally with saline (control) or lipopolysaccharide (LPS 10 mg/kg) and thus an in vivo hypercytokinemia state was simulated. Twenty-four hours after drug injection, indicated organs were collected and then subjected to western blotting as described in the “Methods” section. LPS induced inducible nitric oxide synthase (iNOS) in the heart, kidneys, liver, and lungs, which indicated that inflammatory signal pathways were activated within vital organs. Downstream indicators of activated apoptosis, such as cleaved PARP and cleaved caspase 7, were present in the liver and lungs. Phosphorylation of histone H2AX, an indicator of DNA instability, was also observed in the liver and lungs. Positive bands of cleaved caspase 7, cleaved PARP, and phosphorylated histone H2AX (p-H2AX) were confirmed comparing with the positive controls from doxorubicin (dox)-treated rat H9c2 cells. ctrl; control, N.D.; not determined. (TIFF 2927 kb)

## Abbreviations

ALI: Acute lung injury
BSA: Bovine serum albumin
cox-2: Cyclooxygenase-2
DMSO: Dimethyl sulfoxide
HO-1: Heme oxygenase-1
ICAM-1: Intracellular adhesion molecule-1
iNOS: Inducible nitric oxide synthase
IκB: Inhibitor kappa B
LDH: Lactate dehydrogenase
LPS: Lipopolysaccharide
NF-κB: Nuclear factor kappa B
PARP: Poly ADP-ribose polymerase
