# Supplementary figures and images for: Synergistic cytoprotection by co-treatment with dexamethasone and rapamycin against proinflammatory cytokine-induced alveolar epithelial cell injury
Source: J Intensive Care. 2019 Feb 7;7:12. doi: 10.1186/s40560-019-0365-5 (PMC6367811; doi:10.1186/s40560-019-0365-5)

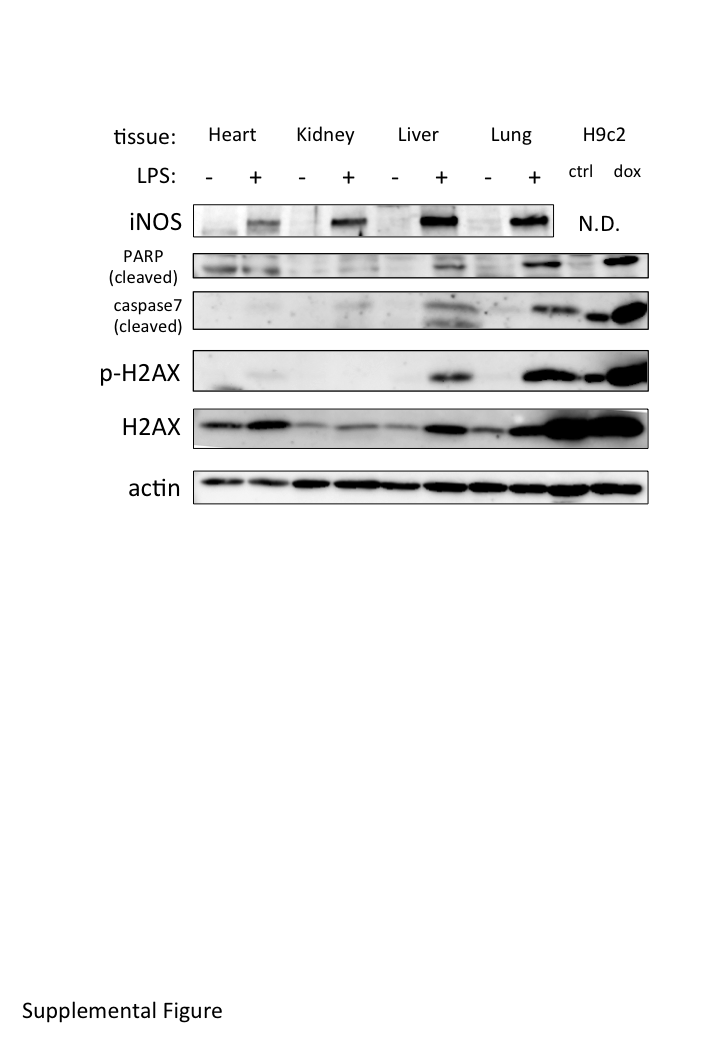

Supplement: Supplementary file 1 — Figure S1. Changes of inflammation-associated protein expression in an endotoxemic mouse. Male C57BL/6 mice (10 weeks) were challenged intraperitoneally with saline (control) or lipopolysaccharide (LPS 10 mg/kg) and thus an in vivo hypercytokinemia state was simulated. Twenty-four hours after drug injection, indicated organs were collected and then subjected to western blotting as described in the “Methods” section. LPS induced inducible nitric oxide synthase (iNOS) in the heart, kidneys, liver, and lungs, which indicated that inflammatory signal pathways were activated within vital organs. Downstream indicators of activated apoptosis, such as cleaved PARP and cleaved caspase 7, were present in the liver and lungs. Phosphorylation of histone H2AX, an indicator of DNA instability, was also observed in the liver and lungs. Positive bands of cleaved caspase 7, cleaved PARP, and phosphorylated histone H2AX (p-H2AX) were confirmed comparing with the positive controls from doxorubicin (dox)-treated rat H9c2 cells. ctrl; control, N.D.; not determined. (TIFF 2927 kb) [file 40560_2019_365_MOESM1_ESM.tiff]
